# Identifying critical gaps in research to advance global surgery by 2030: a systematic mapping review

**DOI:** 10.1186/s12913-023-09973-9

**Published:** 2023-09-04

**Authors:** Meskerem Aleka Kebede, Deng Simon Garang Tor, Tesfamariam Aklilu, Adane Petros, Martilord Ifeanyichi, Ezekiel Aderaw, Maeve Sophia Bognini, Darshita Singh, Rosemary Emodi, Rachel Hargest, Rocco Friebel

**Affiliations:** 1 Global Surgery Policy Unit, LSE Health, London School of Economics and Political Science, Cowdray House 1.12, Houghton Street, London, WC2A 2AE UK; 2 School of Medicine, Addis Ababa University, Addis Ababa, Ethiopia; 3 Royal College of Surgeons of England, Global Affairs, 38-43 Lincoln’s Inn Fields, London, UK; 4 School of Medicine, Cardiff University, Neuadd Meirionnydd, Cardiff, UK

**Keywords:** Global surgery, Network analysis, Equity, World health organization health systems building blocks, Low and middle-income countries, Lancet commission on global surgery, Research funding, Partnership

## Abstract

Progress on surgical system strengthening has been slow due to a disconnect between evidence generation and the information required for effective policymaking. This systematic mapping review sought to assess critical research gaps in the field of global surgery guided by the World Health Organisation Health Systems building block framework, analysis of authorship and funding patterns, and an exploration of emerging research partnership networks. Literature was systematically mapped to identify, screen, and synthesize results of publications in the global surgery field between 2015 and March 2022. We searched four databases and included literature published in seven languages. A social network analysis determined the network attributes of research institutions and their transient relationships in shaping the global surgery research agenda. We identified 2,298 relevant studies out of 92,720 unique articles searched. Research output increased from 453 in 2015-16 to 552 in 2021-22, largely due to literature on Covid-19 impacts on surgery. Sub-Saharan Africa (792/2298) and South Asia (331/2298) were the most studied regions, although high-income countries represented a disproportionate number of first (42%) and last (43%) authors. Service delivery received the most attention, including the surgical burden and quality and safety of services, followed by capacity-building efforts in low- and middle-income countries. Critical research in economics and financing, essential infrastructure and supplies, and surgical leadership necessary to guide policy decisions at the country level were lacking. Global surgical systems remain largely under-researched. Knowledge diffusion requires an emphasis on developing sustainable research partnerships and capacity across low- and middle-income countries. A renewed focus must be given to equipping countries with tools for effective decision-making to enhance investments in high-quality surgical services.

**Supplementary Information:**

The online version contains supplementary material available at 10.1186/s12913-023-09973-9.

## Introduction

Surgical care has evolved from the ‘neglected stepchild’ [1] of global health to occupying an increasingly prominent position in the global health space. The 2015 Lancet Commission on Global Surgery (LCGS) brought surgery onto the international policy agenda, generating momentum for the development of strategies to address the unmet surgical needs of billions of people, most of whom reside in low- and middle-income countries (LMICs) [2]. In addition to the direct health benefits, there is a compelling economic case for strengthening surgical services [3]. Investments in essential surgery can avert an estimated 7.2 million disability-adjusted life years per year [4], and prevent a projected productivity loss of approximately US$12 trillion in LMICs between 2015 and 2030 [2]. The LCGS identified six indicators spanning three domains (i.e., access to timely essential surgery, specialist workforce density, surgical volume, perioperative mortality rate, protection against impoverishing health expenditure, and protection against catastrophic expenditure) to track countries’ progress toward achieving global targets. As policy instruments for mobilizing and streamlining efforts and resources towards scaling-up surgical care at the system level, LMICs were recommended to develop and implement National Surgical, Obstetric, and Anaesthesia Plans (NSOAPs) structured to mirror the World Health Organisation (WHO) health system building blocks [5]. However, for these investments to yield maximal population benefits and meaningful progress to be made, they must be evidence-based [6].

The global surgery momentum has led to a transformation of the discipline from intermittent, short-lived, north-to-south clinical missions driven mainly by non-governmental organizations, to a vibrant academic field. Since 2015, academic global surgery has emerged in the global health lexicon, [7] global surgery (research) centres, education programs, and training opportunities have expanded significantly, and there has been growing interest among surgical trainees to engage in global surgery and international electives [8, 9]. This trend has resulted in an increase in research activities, as demonstrated by a surge in peer-reviewed publications on global surgery [10, 11]. However, despite this progress, many scholars argue that most countries are not on track to achieve the LCGS ambitions for strong surgical systems by 2030. Several reasons have been cited for this, including the Covid-19 pandemic, [12] lack of political commitment from leaders, [13] the dominance of the field by actors from high-income countries (HICs), [14] and misalignment with in-country health priorities [15, 16].

A comprehensive understanding of the challenges impeding global progress and the necessary remedial measure in global surgery requires rigorous knowledge of the evidence base that informs global surgical plans and strategies. Previously conducted studies focussed on different aspects of global surgery scholarship, such as the use of the term ‘global surgery’ in published literature, [10] an assessment of authorship demographics, [17] a systematic review of north-south training partnerships in surgery, [18] a landscape analysis of academic global surgery programs, [7] a scoping review and taxonomy of global surgery education and training, [19] and a mapping of global surgery education in Europe [20]. However, an assessment of the current gaps in the overall global surgery research agenda and its potential drivers remains unknown, despite its importance for the development of impactful scholarship to drive policy and practice and ultimately strengthen surgical systems. Diverse, multidisciplinary, and robust evidence production has been suggested as one of the key pillars for advancement in the field of surgery to achieve ambitious goals by 2030 [2, 21]. Hence, this review sought to explore critical research gaps in the field of global surgery, determine authorship and funding patterns and explore emerging research partnership networks. It provides a comprehensive assessment of existing research to identify critical gaps that require attention by the academic and clinical community to improve knowledge production and ultimately achieve the LCGS 2030 targets.

## Methods

### Search strategy, screening, and data extraction

We performed a systematic mapping review of publications in global surgery since the publication of the LCGS in 2015 in compliance with the Preferred Reporting Items for Systematic Reviews and Meta-Analyses (PRISMA) guideline [22]. A search strategy was designed to encompass all literature within the scope of global surgery and to reflect on diverse terminologies that could be utilized by authors. We used the key concepts ‘low- and middle-income countries’ and ‘surgery’ guided by a health policy information specialist (AF). We focussed on literature from LMICs based on the 2015 World Bank classification [23]. We systematically searched the databases Medline, Embase, Global Health, and Ebsco CINHAL on 24th May 2022 (see search strategy in supplementary file 1).

Deduplication was performed in Endnote. Nine researchers double screened the titles and abstracts of all articles guided by the pre-specified inclusion and exclusion criteria (see inclusion and exclusion criteria in supplementary file 1). Our primary interest was in studies written from a health system perspective. We excluded articles focussed exclusively on HICs, discussing purely clinical aspects of surgery, systematic reviews, scoping reviews, and those without empirical analysis, including opinion pieces, viewpoints, and perspectives. We included studies with a global focus, and those discussing HIC and LMIC collaboration for different aspects of global surgery. We included articles in English, Italian, French, German, Russian, and Mandarin. We used Rayyan [24] for the screening process. Any conflicts between reviewers were addressed via discussion and resolved by a third reviewer when a consensus was not reached. Seven researchers conducted the full text reviewing of articles using double-blinded screening and application of the pre-defined inclusion/exclusion criteria. We ensured 50% overlap between reviewers during coding to ensure consistency.

A customized, pre-tested data collection excel template (see supplementary file 2) was used for data extraction. The information included publication characteristics (title, journal, year, authors, and their affiliations), study setting (country, World Bank and WHO regional categories), research funding institutions, study design, surgical sub-specialty studied (i.e., based on surgical specialities recognised by the American College of Surgeons and Anaesthesia [25]), and the subject of study. When authors were affiliated with more than one country with different World Bank income categorizations, we used the primary affiliation for our analysis.

### Data analysis and reporting

We developed a coding guide based on six thematic groups, corresponding to the six WHO health system building blocks (i.e., health service delivery, health workforce, financing, access to essential medicines, health information system, and leadership/governance) and the LCGs indicators as a proxy for the field’s progress towards achieving 2030 targets. Two of the authors (MAK & RF) pilot coded 50 articles at random and refined the coding guide to reflect the newly emergent themes. The team then deductively coded each included article. We analysed clusters and networks of co-authorship patterns in the field of global surgery using network analysis software (i.e., GEPHI), including authors with a minimum threshold of five publications and articles with up to 20 co-authors (see a detailed analysis included in supplementary file 3).

Moreover, we identified research funding organizations (RFOs) by manually searching funding statements. When there was more than one funder, we recorded all mentioned funders. We then counted the frequency of funding global surgery research wholly or partially by a RFO to identify major research funding agencies and examined the top ten funders based on frequency, their area of research, and geographies.

This study is registered with the Open Science Framework: 10.17605/OSF.IO/EBUPY.

### Ethics statement

The study did not require ethical approval as it was based on published data.

## Results

We identified a total of 117,366 articles published between 2015 and 2022. Following deduplication, 92,720 articles were screened with 2,298 articles included for data extraction. Further details are specified in the PRISMA study flow diagram in supplementary file 1).

Most studies employed quantitative research methodologies, including descriptive studies (n = 1586; 68·9%), cohort studies (n = 122; 5·3%), modelling studies such as economic evaluations (n = 120; 5·2%), quasi-experimental study designs (n = 89; 3·9%), randomized control trials (n = 28; 1·2%),, , and case-control studies (n = 10; 0·5%). A total of 168 qualitative studies were published in the study period, including 13 consensus-building studies (i.e., Delphi) and 30 case reviews. We identified 93 articles employing a mixed-methods approach.

Most research focussed on one single surgical specialty (n = 1645; 71·6%), including general surgery (n = 409; 17·8%), orthopaedics and trauma (n = 201; 8·8%), and obstetrics and gynaecology (n = 197; 8·6%). All other studies focussed on surgery care more generally or covered multiple specialty areas. (A complete list of target fields of study can be found in supplementary file 1).

We mapped the research focus of all studies according to the WHO health system building blocks and pre-specified sub themes (see Table 1). The most investigated aspect of surgical systems was service delivery (n = 1055), followed by surgical workforce (n = 764), and surgical economics and financing (n = 383), with other input categories being less commonly reported. Among the identified sub-themes, studies aiming to assess the burden of the surgical need, surgical volume, and unmet need saw the most research activity. Similarly, studies on surgical education, training, and international collaborations for training were most common within the broader theme of the surgical workforce.

**Table 1:** Research output categories according to the WHO building blocks and related subthemes

WHO health system building block	Sub-theme	Frequency (number)
**Leadership and governance (n = 81)**	Surgical care organisation	23
	Policy planning & implementation (Includes articles discussing NSOAP planning and implementation)	19
	Advocacy & networks	12
	Regulation & accountability	11
	Surgical leadership	9
	Priority setting	7
**Service delivery (n = 1055)**	Quality	335
	Burden, surgical volume, unmet need	437
	Utilization of services (demand & patient-related factors)	146
	International service delivery, short-term mission surgeries	134
	Access	97
	Equity (gender equity included)	64
	Referral	25
**Health workforce (n = 764)**	Surgical education and training	311
	North-south collaborations for training and knowledge exchange	150
	Workforce density	89
	Burnout & satisfaction	80
	Knowledge	77
	Task-shifting/sharing	53
	Gender equity	28
	Attrition/retention/migration	21
	South-south collaborations	7
**Surgical financing (n = 383)**	Costing studies	137
	Economic evaluations	118
	Economic burden on surgical patients	96
	Efficiency/resource use optimization	30
	Provider payment	14
	International funding for surgery including aid	7
	Domestic funding for surgery	1
**Essential medicine and technology (n = 191)**	Supply of essential medicines and equipment	80
	Infrastructure and related factors	61
	Technology and innovation	82
**Health information (n = 95)**	Audits (quality/registry)	46
	Digital information systems	12
	Research	41

We observed a high number of articles assessing surgical volume and burden of disease, and surgical care quality whose frequency is driven by studies concentrating on perioperative mortality. While there was less emphasis on surgical economics and financing, sub-categorisation revealed that the economic burden of surgically treatable illnesses on patients had received modest attention from scholars within the field. There are some areas with disproportionately less research activity. These include research work on leadership and governance, particularly advocacy and networks, the regulation of facilities and workforce, and priority setting. Research on south-south collaborations for training and knowledge exchange had less prominence in the literature. Even though we identified a significant number (n = 118) of economic evaluation studies, there was less emphasis on funding for surgery, whether international or domestic government funding and considerations around surgical provider payment. Only a few studies reported on the role of surgical information systems, particularly digital information systems.

### Geographical distribution

Most research outputs were centred around surgical care in a single LMIC (n = 1962; 85·4%), while a small number of articles focussed on multi-country research (n = 331; 4·45%), with few studies presenting a global focus (n = 41; 1·8%). Among the single-country studies, India (n = 198; 8·65%), Brazil (n = 167; 7·3%), and China (n = 128; 5·6%) were the most researched. However, many identified studies were concentrated in WHO AFRO (n = 775; 34·86%). We identified 496 (22·6%) articles with a focus on surgical care in low-income countries ,668 (30·45%) in lower-middle-income countries, and 720 (38·2%) in upper middle-income countries, according to the World Bank income categories. (A detailed map illustrating study countries can be found in supplementary file 1).

### Authorship and funding

Authors from six countries accounted for more than half (51·3%) of all first authorship across all identified articles. These included affiliations to institutions in the United States of America (26·8%), India (7·7%), Brazil (6·6%), China (5·5%), South Africa (4·8%), and the United Kingdom (4·5%). We found that 41·6% of the lead authors and 40·7% of the last authors (senior authors) were affiliated with institutions in HICs. While 54·3% of the included studies had a lead author affiliated with an institution in the respective study country, 45·6% of the studies had no in-country authorship.

**Fig. 1:**
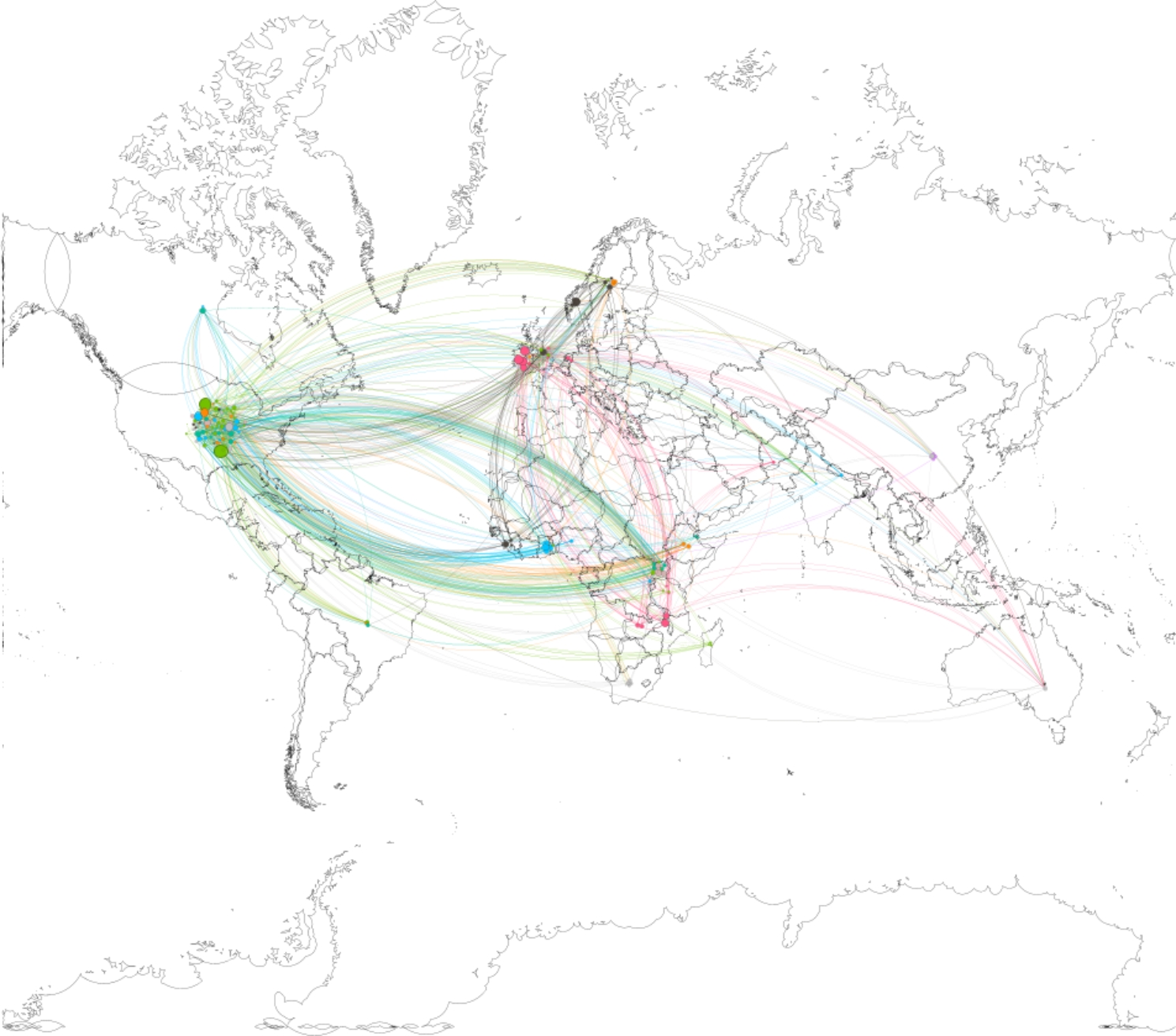
Network analysis of authorship collaborations globally

Our network analysis presented graphically in Fig. 1 suggested geographical clustering around select authors who have had a particular influence on the field of global surgery, which can be used as a proxy to indicate research collaborations. We find frequent collaboration amongst United States of America, European, and United Kingdom based higher education institutions (mostly global surgery units) with researchers based mostly in Africa (i.e., Ghana, Sierra Leone, Tanzania, Ethiopia, and Uganda) (see supplementary file 3 for detailed nodes, edges and network statistics).

**Fig. 2:**
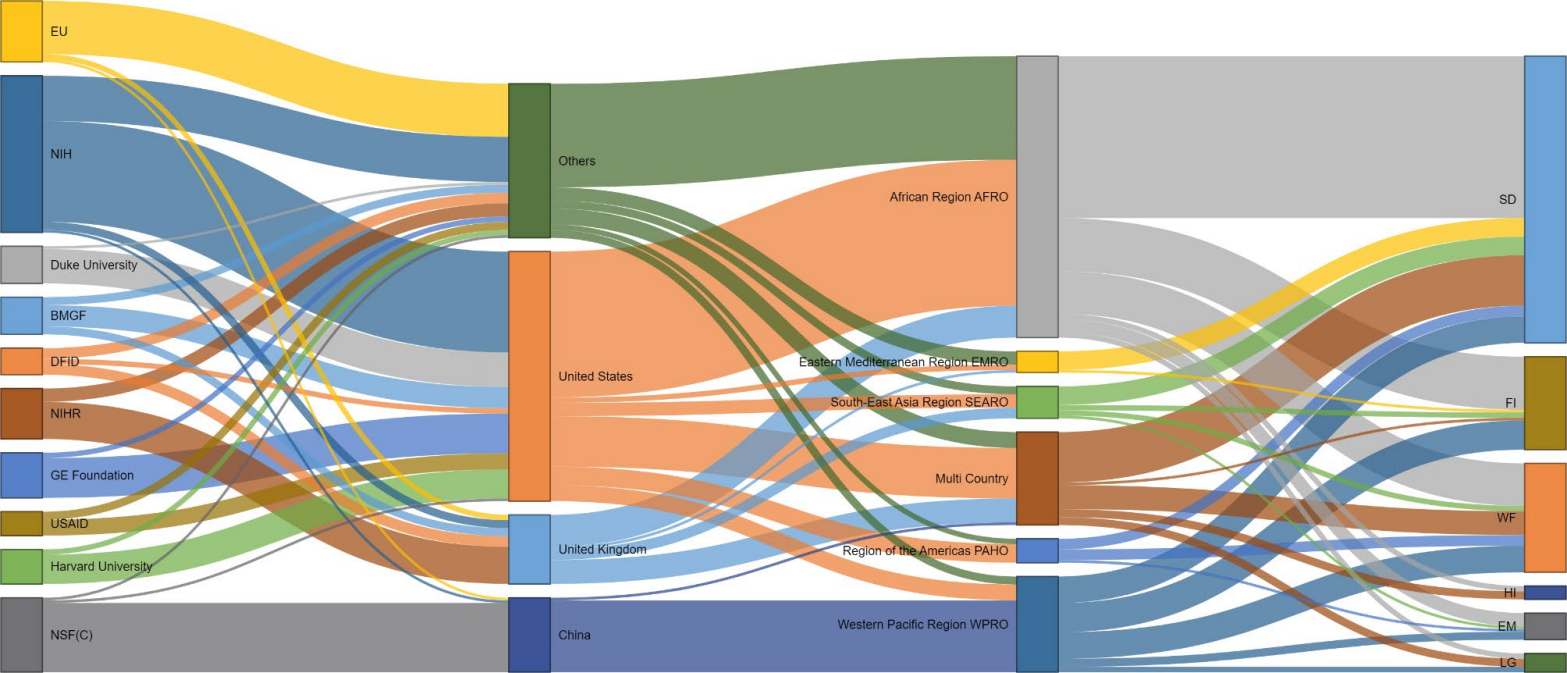
Financial flows of research funds by research funding organisation, country, and research area. Note: EU (European Unioin); NIH (National Institute of Helath); BMGF ( Bill & Mellnda Gates Foundation); DFID (Department for International Development); NIHR (National Institute of Health Research); GE Foundation (General Electric Foundation); USAID (United States Agency for International Development); NSF(c) (National Science Foundation China); SD (Service Delivery); FI(Financing); WF(WorkForce); HI(Health Information); EM(Esential Medicines & Drugs); LG (Leadership & Governance)

Most studies declared no specific funding support (n = 1650; 72·8%). For those that provided funding information (n = 648, 28·2%), we identified 350 distinct RFOs. More than half of the studies were supported by RFOs based in three countries (United States of America, United Kingdom, and China), with the largest proportion of funding channelled through United States of America based research leads, and for research work conducted in sub-Saharan Africa. The second largest share of research funding came from the National Science Foundation China (NSF(c)), whose outputs went almost entirely into research studies with a focus on surgery in China (see Fig. 2 for detailed financial flow information).

### Impact of the Covid-19 pandemic on research in the field of global surgery

The Covid-19 pandemic had a significant impact on the research output, making up 15% (n = 66), 17% (n = 84) and 39% (n = 25) of all studies in 2020, 2021 and 2022, respectively. Articles can be grouped broadly into those describing the impact of Covid-19 on surgical care (e.g., impact on service delivery, on health and wellbeing of the surgical provider workforce, and on surgical education and training) and those describing mitigation strategies (e.g., virtual learning and telemedicine, or reorganizing care pathways and processes).

## Discussion

This study provides a comprehensive assessment of the evidence base guiding policy decision-making toward achieving the 2030 targets set by the LCGS. We find only modest growth in the publication of research studies between 2015 and 2022, though this trend was accelerated by research on the impact and mitigation of the Covid-19 pandemic.

We identify broad alignment between the research areas of the included studies when compared with the indicators proposed by the 2015 LCGS. This finding suggests that the formulation of these indicators likely had an impact on guiding research efforts required to advance the field of global surgery. For example, this is reflected by the consistent increase in publications pertaining to surgical service delivery (burden and unmet need, quality of care, surgical volume, and service uptake and utilization) and workforce (surgical education and training). However, we identify critical research gaps in areas of leadership and governance (priority setting, advocacy networks, accountability, regulation, and surgical leadership), financing (funding for surgery), workforce (south-south collaboration for knowledge transfer), and digital information systems for surgery. It is these gaps in knowledge that have previously been implicated in hindering countries from developing surgical system capacity locally, [10, 26] hampering potential improvements in population health [27].

Proposals for strengthening global surgery called for a comprehensive approach to surgery, spanning across all WHO health system building blocks. The NSOAP, suggested as a key policy framework to guide the incorporation of surgery and anaesthesia into national health plans, identifies six components by contextualizing the WHO health systems framework for surgery [28]. However, we show that authors most frequently publish in few select topic areas. While there are several drivers that could explain this finding, it may be due to the priorities of researchers based outside the study country setting, [29] the relative ease of conducting research in certain topic areas, and the remits and scope set by funders [30]. The clustering of collaborative networks around a few institutions, particularly around medical schools and surgical personnel, might further limit the contribution from multidisciplinary and multisectoral input that is needed to advance global surgery research [31, 32].

Despite surgery being a critical component of healthcare systems, with a strong and resilient surgical system being essential to achieving Sustainable Development Goals 1, 3, 5, 8, 9, 10, 16, 17 and Universal Health Coverage, [33] we note that only a small proportion of global surgery research is funded. Our findings highlight the positive role of grants committed by RFOs based in the United States of America, United Kingdom, European Union, and China in catalysing research in the field. Yet, most funding is channelled to authors based within these regions, with only 0.2% of funding allocated to low-income countries where need is the greatest. Along with an uneven distribution of research authorship favouring researchers based in high-income settings, this counters the goal of equity in academic partnerships, knowledge production, and dissemination in global health with implications for the uptake of findings into policy and practice and research priority identification. This finding aligns with previous work that has indicated the role of RFOs in addressing inequitable knowledge production and equitable partnerships between HICs and their LMIC partners [27, 34–36].

### Strengths and limitations

This is the first comprehensive review of research in the field of global surgery since the publication of the 2015 LCGS. We employed broad selection criteria to capture all research produced in global surgery across four databases, which addressed the limitations of previously conducted reviews [10, 26, 31]. Although the methodological approach aimed at identifying all research studies, including those published in seven languages to address language bias, there may be studies that have not been captured. Limiting our review to indexed research potentially discounted evidence produced through alternative channels, such as reports produced by key global surgery stakeholders and non-governmental organisations active in the field.

We restricted our review to research published on topics of surgery in LMICs. This decision was partly based on the disproportionate surgical need present in these contexts, [4] and the requirement for policymakers to strengthen surgical systems to achieve Universal Health Coverage. However, it also reflected the relative resource constraint of the research team. We acknowledge that a comparison to research output on surgical systems in HICs may be beneficial to aid the understanding of the effectiveness of health system interventions transferrable to lower resourced settings. This includes the focus on technology-driven workforce development solutions, as well as the allocation of resources to improve access, quality, and outcomes of surgical care. Concurrently, this approach would also aid the transferability of knowledge from LMICs to support health systems in HICs that face similar issues, albeit at different scales. Moreover, our results also identified many studies from China and Brazil, which might limit the generalisability to other LMICs and geographic locations.

### Policy recommendations and conclusion

The current trajectory of global surgery research reveals critical gaps in aspects crucial for the strengthening of surgical systems. These research gaps may likely be a consequence of a combination of factors, including the misalignment of research interest and need, lack of multidisciplinary research expertise, and a scarcity of domestic and regional funding for global surgery research in LMICs. Interestingly, there is a growing role of research funding provided by middle-income countries, particularly the NSF(c). A diversified funding landscape may impact on research priorities in the future, possibly with focus on areas that are more closely aligned with LMIC needs to inform policy and practice.

Addressing the unmet need for research requires a multipronged approach from private and public RFOs and academic institutions in countries across income groups to incentivise collaborative research by making funding available and evaluating their impact on promoting and shaping policies. Global health research funders ought to closely examine “capacity building initiatives” built into many grant applications and opt for a more robust monitoring and evaluation practice, with academic journals being intentional in promoting research from underrepresented groups and geographies.

In 2015, the LCGS contributed greatly to the first major momentum to enhance surgery as an essential area of health system strengthening with compelling population health and economic benefits. However, progress on the implementation of actionable policies to improve access to quality surgical services around the world has been slow. More research on leadership, priority setting, and financing of surgical services will be needed to provide the evidence base required by policymakers to prioritise the strengthening of surgical systems.

### Electronic supplementary material

Below is the link to the electronic supplementary material.

Supplementary Material 1

Supplementary Material 2

Supplementary Material 3

Supplementary Material 4

## Data Availability

The datasets used and/or analysed during the current study available from the corresponding author on reasonable request.
